# HIV Infection among Men Who Have Sex with Men in Kampala, Uganda–A Respondent Driven Sampling Survey

**DOI:** 10.1371/journal.pone.0038143

**Published:** 2012-05-31

**Authors:** Wolfgang Hladik, Joseph Barker, John M. Ssenkusu, Alex Opio, Jordan W. Tappero, Avi Hakim, David Serwadda, for the Crane Survey Group

**Affiliations:** 1 Division of Global HIV/AIDS, Center for Global Health, Centers for Disease Control and Prevention (CDC), Entebbe, Uganda; 2 Department of Clinical Epidemiology, Academic Medical Center, University of Amsterdam, Amsterdam, The Netherlands; 3 Ministry of Health, Kampala, Uganda; 4 Division of Global HIV/AIDS, Center for Global Health, Centers for Disease Control and Prevention (CDC), Atlanta, Georgia, United States of America; 5 School of Public Health, Makerere University, Kampala, Uganda; U.S. Naval Medical Research Unit Six (NAMRU-6), Peru

## Abstract

**Background:**

Uganda's generalized HIV epidemic is well described, including an estimated adult male HIV prevalence in Kampala of 4.5%, but no data are available on the prevalence of and risk factors for HIV infection among men who have sex with men (MSM).

**Methodology/Principal Findings:**

From May 2008 to February 2009, we used respondent-driven sampling to recruit MSM ≥18 years old in Kampala who reported anal sex with another man in the previous three months. We collected demographic and HIV-related behavioral data through audio computer-assisted self-administered interviews. Laboratory testing included biomarkers for HIV and other sexually transmitted infections. We obtained population estimates adjusted for the non-random sampling frame using RDSAT and STATA. 300 MSM were surveyed over 11 waves; median age was 25 years (interquartile range, 21–29 years). Overall HIV prevalence was 13.7% (95% confidence interval [CI] 7.9%–20.1%), and was higher among MSM ≥25 years (22.4%) than among MSM aged 18–24 years (3.9%, odds ratio [OR] 5.69, 95% CI 2.02–16.02). In multivariate analysis, MSM ≥25 years (adjusted OR [aOR] 4.32, 95% CI 1.33–13.98) and those reporting ever having been exposed to homophobic abuse (verbal, moral, sexual, or physical abuse; aOR 5.38, 95% CI 1.95–14.79) were significantly more likely to be HIV infected.

**Conclusions/Significance:**

MSM in Kampala are at substantially higher risk for HIV than the general adult male population. MSM reporting a lifetime history of homophobic abuse are at increased risk of being HIV infected. Legal challenges and stigma must be overcome to provide access to tailored HIV prevention and care services.

## Introduction

Men who have sex with men (MSM) are a recognized high-risk group for infection with human immunodeficiency virus (HIV) in the industrialized world. MSM are also well described in select low- and medium-income countries such as Brazil and Thailand [1]. Although HIV epidemics have severely affected large parts of Africa for decades, only recently has there been attention to its spread among African MSM outside of South Africa [2], [3], [4], [5]. Even in the generalized HIV epidemics of sub-Saharan Africa, MSM are three to four times more likely to be HIV infected than the general adult population [6]. MSM in Africa frequently report bi-sexual, often concurrent, relationships, making their female partners an important, and often unwitting, bridging population [7]. Although reliable MSM population size estimates in Africa are largely lacking, the high HIV prevalence observed in several studies would suffice to account for a significant proportion of all new HIV infections in many of these generalized epidemics [4]. Despite this, tailored research for MSM in Africa, especially in HIV prevention, remains an unmet priority [4]. Illegality of homosexual behavior, human rights abuses, and severe stigma add to the specifics' of this continent's HIV epidemic among MSM [2], [8]. As a result, many national HIV policies, AIDS control programs, and HIV services either ignore or inadequately address the specific needs of this most-at-risk population (MARP) in Africa [8]. In Uganda too, public health programming is also impeded due to the illegality of homosexual behavior and the related widespread stigma [6]. An anti-homosexuality bill recently re-proposed in the Ugandan parliament prescribes life-long imprisonment and possibly, including for HIV-infected MSM, the death penalty [9].

Uganda faces a mature, generalized HIV epidemic with an estimated prevalence among the general adult population of 6.4% [10]. HIV infection is more frequent in urban (10.1%) than in rural (5.7%) Ugandan adults, and is higher among female (7.5%) than male adults (5.0%) [10]. Reliable data on the burden of disease among populations at higher risk for HIV are scarce. A recent survey estimated the HIV prevalence among female sex workers (FSW) in Kampala as 33% and that of clients and spouses of FSW as 18% [11]. An additional high risk group in Uganda includes mobile men with money, such as fishermen among whom a recent survey found an HIV prevalence of 16.4% [12].

For many hard-to-reach populations, including MSM, respondent-driven sampling (RDS) can be an effective sampling tool that facilitates population-based, weighted estimates. [13], [14], [15]. RDS-based MSM surveys have been conducted in numerous countries [16], including in Africa. A survey conducted in 2004 in Kampala confirmed the existence of an indigenous MSM population and the presence of HIV-related risk behaviors, including high rates of commercial or transactional sex [17]. We report here on a more recent survey, the Crane Survey, that aimed at estimating the burden of HIV and STI in select groups assumed to be at increased risk for HIV. While our survey among Kampala MSM included for the first time testing for both HIV and STI, we report here on the HIV-related findings; separate analyses examine non-HIV domains (including STIs and behavioral outcomes) in more detail.

## Methods

### Ethics statement

The survey protocol and consent procedures were approved by the Uganda Virus Research Institute's institutional review board, the Uganda National Council of Science and Technology, and the CDC. The survey was conducted anonymously; informed consent was obtained verbally; no personal identifiers were collected.

### Survey design

This survey was cross-sectional and used RDS. The survey is part of the “Crane Survey”, a joint activity by Makerere University, the Ministry of Health (MoH), and the US Centers for Disease Control and Prevention (CDC). Prior to the survey, formative research was carried out over several weeks through approximately a dozen key informant interviews using semi-structured interview guides or open discussions to inform sampling design, seed identification, social connectedness among MSM in Kampala, number and location of survey offices, protection of privacy, compensation, data and biological measures, language, and other procedures. Key findings included that RDS was recommended and preferred over time-location sampling, that a single survey office would suffice, that anonymity was paramount, and that biomarker results needed to be returned and addressed. We conducted the survey between May 2008 and April 2009.

### Setting

The survey was carried out in Kampala, Uganda's capital and largest city with approximately 1.8 million residents.

### Study population

Inclusion criteria were male sex, age ≥18 years, residence in greater Kampala, and self-reported anal sex with another man in the preceding 3 months. Exclusion criteria included coupon receipt from a stranger and language barriers.

### Sampling

RDS methodology is well described elsewhere [13], [14], [15] and represents an advanced version of chain referral sampling. We initiated sampling with eight seeds purposively selected by age, HIV status, and geographic location in Kampala; all were socially well networked. After seeds began their recruiting efforts, candidate recruits presented the coupons they had received to the single survey office in Old Kampala, near the city center. Sampling was interrupted twice; first as a result of the arrest of three local lesbian, gay, bisexual, and transgender (LGBT) activists at the PEPFAR Implementer's Meeting in Kampala in early June 2008 [18]. Sampling remained at a low level until it rebounded in August, but was again affected in September 2008 by arrests of alleged homosexuals [19]. A total of six additional seeds were added during the survey. However, following the second round of arrests, sampling rates remained low until we stopped sampling in March 2009, close to the survey's scheduled end.

We issued 1,706 coupons and redeemed 455 of these. The number of coupons issued per recruit ranged from two at the survey's beginning to six after the arrests and slump in sampling. A total of 300 eligible recruits (n = 286) and seeds (n = 14) participated in the survey; the remainder were ineligible, mostly because they did not report anal sex with men in the last 3 months. The longest recruitment wave was 11; equilibrium for HIV serostatus was reached after wave number 2, and that for nationality, age, and condom use at last sex was reached after wave number 3, 4, and 4, respectively, the *computed* design effect (using actual survey data) for HIV infection was 2.3.

### Survey office procedures

Candidate participants were screened for eligibility face-to-face by survey staff. Following a briefing about specific interview terms, such as the definition of sexual intercourse, frequency of sex, partner types, or commercial sex and a short computer-based tutorial about audio-computer-assisted self-interviewing (ACASI), enrolled participants underwent a standardized interview using Questionnaire Design Studio (QDS v2.5) software (NOVA, Bethesda, Maryland, USA). A small number of recruits preferred a computer-assisted personal interview with trained staff using the same QDS instrument. Following the interview and pre-test counseling, recruits provided venous blood and urine samples, and had rectal swabs collected. At the end of the first visit, recruits received instructions and coupons for peer recruitment.

Recruits were scheduled to return to the survey office two weeks later. Survey staff post-test counseled recruits for all biomarkers measured, provided treatment for non-viral sexually transmitted infections (STI) according to World Health Organization and MOH guidelines, and referred HIV-positive recruits to health care providers with whom we had made arrangements prior to survey start. Recruits who failed to return to the survey office could not be reached by survey staff as no personal identifiers were collected. Recruits who returned to the survey office when not all biomarker results were yet available were asked (and compensated for) to return for an additional visit.

At both visits to the survey office, we compensated recruits for their time and transport costs (US$3.00), and, at the return visit, recruitment efforts (US$1.00 per successfully recruited eligible peer). At the time of the survey, US $3.00 could purchase three kilograms of sugar.

### Data measures

The main interview's key domains included demographics, life-time sexual characteristics, sexual behaviors in the last three months, sexual violence, and STDs. Condom use was examined both quantitatively (number of protected sex acts divided by all sex acts in last three months), as well as qualitatively (condom use ever or never, condom use at last sex). Lifetime exposure to homophobic abuse was measured through the question “*Did you ever suffer any violence or abuse because you have sex with other men?*”. Respondents who affirmed such abuse, where probed about the type of abuse, including moral abuse (isolation or exclusion), verbal abuse (threats or insults), mistreatment, or having been subjected to physical or sexual violence. Respondents were also probed about blackmail (“*Have you ever been blackmailed by someone because you have sex with other men?*”) and rape (“*Were you ever forced to have sex against your will?*”). Other data measures included alcohol use, drug use (including injection drug use), as well as buying sex (defined as paying for sex with money, goods or services) or selling sex (defined as giving sex in exchange for money, goods or services).

### Laboratory measures

Laboratory testing was performed off-site at the STD Reference Laboratory based at Mulago Hospital, Kampala; a small number of tests were also performed at the CDC laboratory in Entebbe, Uganda. Blood specimens were stored at 2 to 8°C at the survey office and transported twice daily to the laboratory. Testing for antibodies against HIV was performed through a parallel testing algorithm using Vironostika® HIV Uniform II plus O2 (bioMeriéux, Marcy l'Etoile, France) and Murex® HIV Ag/Ab Combination (Abbott Laboratories, Abbott Park, Illinois, U.S.A.); discordant results were resolved through the use of HIV 1/2 STAT-PAK rapid test (Inverness Medical, Princeton, New Jersey, U.S.A.). All recruits enrolled into the survey accepted HIV testing. Plasma was also tested for *Treponema pallidum* (TP) infection, using the Anti-syphilis IgG ELISA (Biotec Laboratories, Suffolk, UK) for screening and, if reactive, the Rapid Plasma Reagin Syfacard-R Test (Murex Biotech, Dartford, UK) to detect current TP infection. Urine specimens and rectal swabs were tested for the presence of *Neisseria gonnorhea* (NG) and *Chlamydia trachomatis* (CT) DNA (Cobas Amplicor or Amplicor PCR, Roche Diagnostics, Branchburg, New Jersey, U.S.A.).

### Data management and analysis

For sample size calculations, we assumed an HIV prevalence of 14%, 95% confidence intervals (CI) of 10.4%–18.3% (approximately twice that of urban men in general [14]), and a design effect of 2 [15]. Aiming for an effective sample size of 300, we adjusted the target sample size to 600.

Survey events (enrollments, recruiter-recruitee links, coupons numbers issued, unique codes, etc.) were tracked with an in-house developed software. Interview data were checked for errors and inconsistencies, and cleaned after importation from QDS into Statistical Analysis Software - SAS v9.2 (SAS Institute, Gary, North Carolina).

Of the 300 eligible respondents, 5 provided little interview data and were excluded from this analysis. Our principal outcome of interest was HIV infection; predictor variables included demographics, sexual orientation and experience, sexual behavior in the 3 months preceding the interview, HIV testing (history), sexual violence, abuse, or blackmail, as well as laboratory markers of sexually transmitted infections and STD symptoms. Condom use over the last three months includes both male and female sex partners (unless stated otherwise) and was computed as a continuous variable with the denominator being the number of sex acts and the numerator being the number of sex acts protected by condoms. We examined condom use as a categorical variable, indicating whether in the preceding three months condoms were used for less than 33% of all sex acts, for 33%–66% of sex acts, or for more than 66% of sex acts.

We present weighted data except for continuous data; univariate analyses were conducted in RDSAT version 6.0.1. (www.respondentdrivensampling.org). Individual HIV sampling weights were generated in RDSAT and exported to STATA. Using HIV weights imported from RDSAT, logistic regression was conducted for bivariate and multivariate analysis in STATA 10.0 (Stata Corporation, College Station, Texas). For multivariate analysis we employed backward elimination using predictor variables associated with HIV infection at a level of *P*≤0.2 in bivariate analysis. The final weighted model displays all predictor variables significantly associated with HIV infection at a level of *P*≤0.05.

### Human subjects considerations

Using scanners and Griaule software (Griaule Biometrics, San Jose, CA, USA), recruits' fingerprints were imaged (but not stored) to generate unique alphanumeric codes and thus facilitate linking recruits' return visits to their initial visits and laboratory results, to detect duplicate recruits (recruits attempting to enroll multiple times), and to detect recruits presenting coupons that had already been issued to other recruits. In addition to this survey, we sampled four other groups concurrently using the same survey office and hence masked the group identity of any survey respondent visiting in the survey office.

## Results

Of the 295 MSM considered for analysis, no recruit refused survey participation, and no enrolled recruit refused biomarker testing. Sixty-three percent of recruits returned to the survey office and received post-test counseling. Among the 37% who did not return to the survey office and therefore were not post-test counseled 8.5% had an HIV-positive test result. Table 1 shows key characteristics of Kampala MSM. Almost all were of African race and Ugandan nationality; 81% stated their last male sex partner was Ugandan. About half were aged 18–24 years old and fewer than one in five were unemployed. MSM reported a median of 11 years in school and about three quarters had attended secondary school or higher. Three-quarters of Kampala MSM had ever had sex with women, one-third had fathered children, and one-third had ever been married. At the time of the survey, 13.3% were married and 16.4% lived with a female sex partner.

**Table 1:** MSM characteristics, Kampala, Uganda, 2008/9.

	Numerator unweighted (N = 295)	Sample %	Estimated population proportion* % (N = 281)	95% C.I.
**Race**				
African	276	93.9	94.0	91.1–97.4
Other	18	6.1	6.0	2.6–8.9
**Nationality**				
Ugandan	267	91.4	93.9	90.4–97.2
Other	25	8.6	6.1	2.8–9.6
**Religion**				
Catholic	123	42.0	39.2	31.7–47.0
Protestant	73	24.9	29.0	21.8–36.8
Moslem	45	15.4	17.1	10.9–23.3
Other religion	41	14.0	11.6	7.5–15.9
None	11	3.8	3.1	0.6–7.4
**Age (years)**				
18–24	143	48.5	49.7	39.2–56.5
25+	152	51.5	50.3	43.5–60.8
**Schooling (years)**				
0–6	71	24.6	24.9	17.4–32.5
7+	218	75.4	75.1	67.5–82.6
**Occupation**				
Student	52	17.8	18.5	11.1–24.7
Unemployed	49	16.8	16.0	10.6–22.0
Employed	191	65.4	65.5	57.7–74.6
**Marital status (with women)**				
Never married	194	66.0	70.2	61.9–77.6
Married	57	19.4	13.2	8.8–19.1
Previously married	43	14.6	16.5	10.5–22.7
**Current marital status**				
Yes	57	19.4	13.3	9.0–19.3
No	237	80.6	86.7	80.7–91.0
**Heterosexual exposure**				
Ever sex with a woman	226	76.6	77.6	70.7–83.5
Ever fathered children	94	32.2	29.6	21.8–37.1
**Living with a female sex partner**				
Yes	53	18.0	16.4	11.2–22.0
No	241	82.0	83.6	78.0–88.8
**Circumcision status**				
Circumcised	145	49.2	44.1	35.5–53.6
Uncircumcised	150	50.8	55.9	46.4–64.5
**Ever tested for HIV**				
Yes	128	45.1	43.4	36.5–52.2
No	156	54.9	56.6	47.5–63.5
**No. sex partners in last 6 months**				
< = 10 partners	133	48	52.7	44.4–63.8
11–24 partners	57	20.6	23.0	15.1–29.3
> = 25 partners	87	31.4	24.3	16.8–31.5
**STIs**				
Yes	40	13.9	12.4	8.2–17.8
No	248	86.1	87.6	82.2–91.8
**Nationality of last male sex partner**				
Ugandan	225	79.2	81.1	75.5–87.2
Other African country	33	11.6	10.9	6.1–15.8
Outside Africa	23	8.1	6.7	3.3–11.0
Don't know	3	1.1	1.3	0–2.4
**Sexual orientation**				
Gay/homosexual	166	56.8	55.0	48.4–63.3
Bisexual	113	38.7	37.6	29.4–44.1
Heterosexual	13	4.5	7.4	3.0–13.1
**Sexual attraction**				
Mostly/only to men	208	71.0	68.8	62.0–76.7
Equally to men and women	38	13.0	12.3	7.3–15.8
Mostly/only to women	47	16.0	18.8	13.5–25.1

Note: Denominators vary due to missing data (refuse to answer, don't know).

* ) Estimated using RDSAT software.

The estimated weighted HIV prevalence was 13.7% (95% confidence intervals [CI] 7.9%–20.1%). The HIV prevalence among 18–24 year old MSM was estimated at 3.9%, whereas that among 25+ year-old MSM was estimated at 22.4%. .Previous HIV testing was reported by 43.4%, with a median time since last HIV test of 12.5 months (interquartile range 4.9–30.2). A small proportion (3.9%) reported that they had tested HIV-positive; of those who reported a previous HIV-positive result (n = 10) or perceived themselves as HIV-infected (n = 16), only 10% actually tested HIV-positive in our survey. Conversely, of those who tested HIV-positive in our survey, 50% thought of themselves as HIV-negative and most of the remaining reported not knowing their HIV status.

More than one in three (39%) MSM stated having ever suffered homophobic abuse. Types of abuse included moral (including “isolation”, “exclusion” [18.2%]) and verbal (“threats”, “insults” [33.2%]) mistreatment, as well as physical (15.5%) and sexual violence (22.0%). Abuse most frequently originated from family members (25.4%), sex partners (24.2%), and friends and acquaintances (24.1%).

Table 2 shows the results of bi-variate analysis of potential risk factors for HIV infection. MSM aged ≥25 years were more likely to be HIV infected than their younger peers (odds ratio [OR] 5.69, 95% CI 2.02–16.02). Furthermore, MSM who reported ever having been exposed to homophobic abuse or violence were six times as likely to be HIV-infected as their non-abused peers (OR 6.41, 95% CI 2.52–16.35). Also, MSM who reported anal or genital STD symptoms in the previous 12 months had three times the odds of HIV infection compared with MSM who reported neither (OR 3.06, 95% CI 1.19–7.86). A history of illicit drug use, injection drug use, and a history of never using lubricants were paradoxically associated with lower odds for being HIV infected in bivariate analysis; injection drug use and lack of lubricant use, however, lost significance in multivariate analysis. Other predictors that were not significantly associated in the weighted analysis included circumcision status, education, alcohol use, ever using condoms, type of anal sex, engaging in commercial sex, number of lifetime sex partners, and testing positive for other STI.

**Table 2:** Bivariate associations with HIV status.

	Sample estimates		Population estimates		
	HIV− (%)	HIV+ (%)	Odds ratio	95% CI	*p*
**Age (years)**					
18–24	137 (95.8)	6 (4.2)	Ref	-	-
25+	117 (77.5)	34 (22.5)	5.69	2.02–16.02	0.001
**Circumcision status**					
Uncircumcised	131 (87.3)	19 (12.7)	Ref	-	-
Circumcised	123 (85.4)	21 (14.6)	0.84	0.35–2.01	0.690
**No. years in school**					
0–6	64 (90.1)	7 (9.9)	Ref	-	-
7+	185 (85.3)	32 (14.7)	1.55	0.56–4.34	0.401
**Religion**					
Catholic	105 (85.4)	18 (14.6)	Ref	-	-
Protestant	63 (87.5)	9 (12.5)	1.18	0.37–3.78	0.775
Moslem	36 (80.0)	9 (20.0)	1.06	0.37–3.04	0.912
Other religion	37 (90.2)	4 (9.8)	0.38	0.10–1.47	0.161
None	11 (100)	0 (0)	-	-	-
**Occupation**					
Student	50 (96.2)	2 (3.8)	Ref	-	-
Unemployed	43 (87.8)	6 (12.2)	2.42	0.42–13.99	0.322
Employed	158 (83.2)	32 (16.8)	4.03	0.86–18.87	0.077
**Marital status (with women)**					
Never married	168 (86.6)	26 (13.4)	Ref	-	-
Married	53 (94.6)	3 (5.4)	0.21	0.06–0.80	0.021
Previously married	32 (74.4)	11 (25.6)	2.78	0.88–8.77	0.081
**Alcohol consumption past 30 days**					
None	81 (94.2)	5 (5.8)	Ref	-	-
Less than once a week	22 (91.7)	2 (8.3)	0.71	0.09–5.26	0.734
At least once a week	108 (85.0)	19 (15.0)	1.45	0.48–4.37	0.504
About every day	40 (75.5)	13 (24.5)	4.72	1.24–17.96	0.023
**Illict drug consumption**					
Never	174 (82.9)	36 (17.1)	Ref	-	-
Ever	80 (95.2)	4 (4.8)	0.14	0.04–0.54	0.004
**Ever injected drugs**					
Never	223 (85.1)	39 (14.9)	Ref	-	-
Ever	31 (96.9)	1 (3.1)	0.08	0.01–0.60	0.014
**Condom use**					
Never	67 (88.2)	9 (11.8)	Ref	-	-
Ever	179 (86.1)	29 (13.9)	2.00	0.75–5.32	0.167
**Condom use (for sex acts in last 3 months)**					
>66%	91 (86.7)	14 (13.3)	Ref	-	-
33%–66%	50 (84.8)	9 (15.2)	2.28	0.71–7.36	0.167
<33%	98 (88.3)	13 (11.7)	0.59	0.23–1.53	0.281
**Lubricant use**					
Ever	190 (84.1)	36 (15.9)	Ref	-	-
Never	55 (96.5)	2 (3.5)	0.12	0.02–0.67	0.015
**Ever sex with women**					
Never	55 (79.7)	14 (20.3)	Ref	-	-
Ever	199 (88.4)	26 (11.6)	0.65	0.26–1.61	0.354
**History of blackmail**					
Never	116 (92.1)	10 (7.9)	Ref	-	-
Ever	74 (84.1)	14 (15.9)	1.76	0.55–5.61	0.339
**History of homophobic abuse**					
Never	156 (93.4)	11 (6.6)	Ref	-	-
Ever	88 (76.5)	27 (23.5)	6.41	2.52–16.35	<0.001
**History of rape**					
Never	172 (86.4)	27 (13.6)	Ref	-	-
Ever	73 (86.9)	11 (13.1)	0.85	0.34–2.11	0.722
**Type of anal sex (last 3 months)**					
Mostly insertive	110 (90.9)	11 (9.1)	Ref	-	-
Equally insertive & receptive	58 (82.9)	12 (17.1)	1.17	0.35–3.89	0.797
Mostly receptive	63 (82.9)	13 (17.1)	1.64	0.49–5.44	0.419
**Selling sex**					
Never	133 (85.8)	22 (14.2)	Ref	-	-
Ever	113 (87.6)	16 (12.4)	1.42	0.56–3.61	0.463
**Buying sex**					
Never	123 (86.6)	19 (13.4)	Ref	-	-
Ever	110 (89.4)	13 (10.6)	0.91	0.38–2.21	0.842
**No. sex partners in last 6 months**					
< = 10 partners	109 (81.9)	24 (18.1)	Ref	-	-
11–24 partners	51 (89.5)	6 (10.5)	1.01	0.25–4.12	0.986
> = 25 partners	80 (91.9)	7 (8.1)	0.60	0.21–1.70	0.336
**No. life time sex partners**					
< = 10 partners	63 (84.0)	12 (16.0)	Ref	-	-
11–24 partners	60 (89.5)	7 (10.5)	0.68	0.21–2.17	0.512
> = 25 partners	104 (86.7)	16 (13.3)	1.26	0.42–3.73	0.681
**STD symptoms (last 12 months)**					
No	118 (91.5)	11 (8.5)	Ref	-	-
Yes	117 (81.8)	26 (18.2)	3.06	1.19–7.86	0.02
**Treponema pallidum (syphilis)**					
Negative	233 (87.6)	33 (12.4)	Ref	-	-
Positive	21 (75.0)	7 (25.0)	2.56	0.79–8.25	0.115
**Chlamydia trachomatis**					
Negative	245 (86.6)	38 (13.4)	Ref	-	-
Positive	3 (60.0)	2 (40.0)	3.07	0.45–20.80	0.25
**Neisseria gonorrhoea**					
Negative	241 (86.4)	38 (13.6)	Ref	-	-
Positive	7 (77.8)	2 (22.2)	1.77	0.30–10.32	0.527
**HIV testing history**					
Never	138 (88.5)	18 (11.5)	Ref	-	-
Ever	108 (84.4)	20 (15.6)	1.30	0.50–3.34	0.588
**No. sex partners in the last 6 months**					
< = 10 partners	109 (81.9)	24 (18.1)	Ref	-	-
11–24 partners	51 (89.5)	6 (10.5)	1.01	0.25–4.12	0.986
> = 25 partners	80 (91.9)	7 (8.1)	0.60	0.21–1.70	0.336
**Sexual orientation**					
Gay/homosexual	138 (83.6)	27 (16.4)	Ref	-	-
Bisexual	102 (90.3)	11 (9.7)	0.56	0.23–1.35	0.194
Heterosexual	11 (84.6)	2 (15.4)	2.98	0.44–20.2	0.263
**Sexual attraction**					
Mostly/only to men	173 (83.6)	34 (16.4)	Ref	-	-
Equally to men and women	36 (94.7)	2 (5.3)	0.26	0.05–1.30	0.101
Mostly/only to women	44 (93.6)	3 (6.4)	0.30	0.08–1.15	0.080

Table 3 displays the results of the multivariate analysis. After controlling for potential confounders in the weighted multivariate analysis, MSM of older age or reporting a history of homophobic abuse were four and five times as likely to be HIV-infected as their younger or non-abused MSM, respectively. Injection drug use was no longer significantly associated with HIV status and was not included in the final multivariate model. Instead, we examined self-reported drug use, regardless of the mode of application, which was inversely related with HIV seropositivity in multivariate analysis.

**Table 3:** Factors associated with HIV seropositivity among MSM (multivariate weighted analysis).

Characteristic	Unadjusted OR (95% CI)	*p*	Adjusted OR (95% CI)	*p*	
**Age (years)**					
18–24	Ref		Ref		
25+	5.69 (2.02–16.02)	0.001	4.32 (1.33–13.98)	0.015	
**Illict drug consumption**					
Never	Ref		Ref		
Ever	0.14 (0.04–0.54)	0.004	0.15 (0.04–0.65)	0.01	
**History of homophobic abuse**					
Never	Ref		Ref		
Ever	6.41 (2.52–16.35)	<0.001	5.38 (1.95–14.79)	0.001	

Factors non-significant in multivariate analysis but adjusted for: lubricant use, current marital status, sexual orientation, sexual attraction, history of STD symptoms.

## Discussion

This is the first bio-behavioral survey among MSM in Uganda. The weighted HIV prevalence estimate of 13.7% is substantially higher than the 4.5% estimate for general adult men in Kampala [10]; the resulting HIV prevalence ratio of 2.9 seems consistent with those found elsewhere in generalized epidemics [6]. This survey also found that Kampala MSM who suffered homophobic abuse were five times as likely to be HIV-infected as those who escaped such abuse. The estimated 44% of MSM ever having been tested for HIV is substantially higher than the estimated 12% in 2004/5 among Kampala men in general [10]; however, we found poor concordance between recruits' stated or perceived HIV status and their actual status as measured in our survey.

This survey reaffirms some basic facts, namely that almost all MSM in Kampala are Ugandan nationals, dispelling claims that there are no local MSM or that they only have sex with foreigners. Further, most MSM had or still have sex with women, many were or are married, co-habiting with women, and/or have biological children. Thus, MSM in Kampala appear firmly embedded in the general population. The large proportion of MSM that report buying or selling sex and the less frequent reporting of life-time injection drug serve as a reminder that Kampala MSM share risk factors beyond their defining behavioral characteristic.

Sampling proved difficult due to the repeated arrests of LGBT individuals. A previous survey among gay men in Kampala also faced challenges [17], confirming that conducting such surveys can be difficult in the face of criminalization and homophobia. Nevertheless, the widespread misperceptions, falsehoods, and prejudice against MSM in many African countries make empirical data collection all the more relevant. The 2008 Kampala arrests prevented us from achieving the target sample size, and the resulting decreased statistical power may have impeded identifying some risk factors for HIV infection. However, equilibrium was reached for the primary outcome of interest and allowed to describe the burden of HIV disease. RDS is well suited to sample hard-to-reach communities such as Kampala's MSM, however, we cannot rule out the possibility that we missed separate, isolated MSM networks in Kampala. Anecdotal evidence also suggests that our survey staff may have excluded some MSM who were too embarrassed or afraid to indicate their same sex activity during the face-to-face screening interview. Other MSM were excluded because their last same sex encounter occurred more than three months prior to the screening interview; thus our survey represents currently active MSM and excludes men who do not or only infrequently exercise their homosexual orientation. For future surveys we will consider extending the eligibility recall period for same sex. Lastly, methodological challenges in conducting multivariate analysis using RDS-derived data remain and actual design effects in RDS surveys may be substantially larger than estimated with current software packages [20].

MSM reporting illicit drug use were significantly less likely to be HIV-infected that MSM who did not report such abuse, a paradoxical finding. Most drug use among MSM in Kampala is of non-injecting nature. The distribution of potential determinants for HIV infection (age, type of drugs, number of sex partners, number of sex acts, type of sex, condom use) by drug using status did not lend themselves to any interpretation supporting this unexpected finding (data not shown). Older age and exposure to homophobic abuse were strongly associated with HIV infection in multivariate analysis. As a chronic infection, the association between HIV infection and age is not surprising and may reflect the cumulative effect of long-term risk behavior. The cross-sectional nature of our survey design limits our interpretation of cause and effect on the observed association between exposure to homophobic violence or harassment and HIV infection; further, our data instrument did not probe the timing of abuse. Homophobic abuse is also reported in other African countries [2] and its association with HIV infection warrants further research in this region. Existing literature suggests that homophobia, criminalization, and discrimination impede access to HIV prevention services for MSM and increase their vulnerability [21]. Similarly, gay-related physical abuse and harassment are related to HIV infection among gay and bisexual U.S. men [22], and homophobia may contribute to the racial differences in HIV infection rates seen among U.S. MSM [23].

Our survey's finding of the increased risk of HIV infection in Kampala MSM call for their inclusion in strategic plans for HIV prevention, care, and treatment. Renewed attention is warranted to the largely unmet but specific HIV-related prevention needs of MSM in Uganda, including improved access to HIV and STI testing and treatment, MSM-tailored counseling, provision of condoms and safe lubricants, and measures to improve their physical safety and human rights situation. Regardless of the illegality of same sex behavior, Uganda's National Strategic Plan for HIV/AIDS ought to address the public health needs of this high-risk group and facilitate tailored service provision through local non-governmental and community-based organizations. MSM's inadequate access to HIV and STI related services likely contributes significantly to the overall burden of HIV disease in Uganda. This survey also serves as a reminder that Uganda's MSM do not live in isolation, that denying them HIV-related services puts all of the country's citizens at risk and that homophobia may fuel the HIV epidemic in Uganda.

## References

[1] Caceres C, Konda K, Pecheny M, Chatterjee A, Lyerla R (2006). Estimating the number of men who have sex with men in low and middle income countries.. Sex TransmInfect.

[2] Baral S, Trapence G, Motimedi F, Umar E, Iipinge S (2009). HIV prevalence, risks for HIV infection, and human rights among men who have sex with men (MSM) in Malawi, Namibia, and Botswana.. PLoSONE.

[3] Sanders EJ, Graham SM, Okuku HS, van der Elst EM, Muhaari A (2007). HIV-1 infection in high risk men who have sex with men in Mombasa, Kenya.. AIDS.

[4] van GF (2007). Men who have sex with men and their HIV epidemics in Africa.. AIDS.

[5] Wade AS, Kane CT, Diallo PA, Diop AK, Gueye K (2005). HIV infection and sexually transmitted infections among men who have sex with men in Senegal.. AIDS.

[6] Baral S, Sifakis F, Cleghorn F, Beyrer C (2007). Elevated risk for HIV infection among men who have sex with men in low- and middle-income countries 2000–2006: a systematic review.. PLoSMed.

[7] Beyrer C, Baral SD, Walker D, Wirtz AL, Johns B (2010). The expanding epidemics of HIV type 1 among men who have sex with men in low- and middle-income countries: diversity and consistency.. EpidemiolRev.

[8] International Gay and Lesbian Human Rights Commission (IGLHRC, 2007) Africa: Off the Map..

[9] Uganda Anti-Homosexuality Bill.. http://en.wikipedia.org/wiki/Uganda_Anti-Homosexuality_Bill.

[10] (2006). Ministry of Health (MOH) [Uganda] and ORC Macro. Uganda HIV/AIDS Sero-behavioural Survey 2004–2005.

[11] Makerere University, School of Public Health, Kampala, Uganda (2010). The Crane survey report: High risk group surveys conducted in 2008/9, Kampala, Uganda.. https://sites.google.com/site/cranesurvey/downloads.

[12] Ministry of Health (MoH), STD/AIDS Control Programme (2011). HIV/AIDS serobehavioural survey among fishing communities in Uganda..

[13] Heckathorn DD (1997). Respondent-driven sampling: A new approach to the study of hidden populations.. Social Problems.

[14] Heckathorn DD (2004). Extensions of respondent-driven sampling: Analyzing continuous variables and controlling for differential recruitment.. Soc Method.

[15] Heckathorn DD (2007). Respondent-Driven Sampling II: Deriving Valid Population Estimates from Chain-Refferal Samples of Hidden Populations.. Social Problems.

[16] Malekinejad M, Johnston LG, Kendall C, Kerr LR, Rifkin MR (2008). Using respondent-driven sampling methodology for HIV biological and behavioral surveillance in international settings: a systematic review.. AIDS Behav.

[17] Kajubi P, Kamya MR, Raymond HF, Chen S, Rutherford GW (2008). Gay and bisexual men in kampala, Uganda.. AIDS Behav.

[18] International Gay and Lesbian Human Rights Commission (2008). Uganda: LGBT Arrested at International HIV/AIDS Meeting.. http://www.iglhrc.org/cgi-bin/iowa/article/takeaction/partners/227.html.

[19] International Gay and Lesbian Human Rights Commission (2008). Uganda: Demand an end to official harassment of LGBT activists.. http://www.iglhrc.org/cgi-bin/iowa/article/takeaction/partners/203.html.

[20] Goel S, Salganik MJ (2010). Assessing respondent-driven sampling.. Proc Natl Acad Sci U S A.

[21] Beyrer C (2010). Global prevention of HIV infection for neglected populations: men who have sex with men.. ClinInfectDis.

[22] Friedman MS, Marshal MP, Stall R, Cheong J, Wright ER (2008). Gay-related development, early abuse and adult health outcomes among gay males.. AIDS Behav.

[23] Glick SN, Golden MR (2010). Persistence of racial differences in attitudes toward homosexuality in the United States.. J Acquir Immune Defic Syndr.

